# Direct Metagenomic Diagnosis of Community-Acquired Meningitis: State of the Art

**DOI:** 10.3389/fmicb.2022.926240

**Published:** 2022-07-05

**Authors:** Madjid Morsli, Jean Philippe Lavigne, Michel Drancourt

**Affiliations:** ^1^IHU Méditerranée Infection, Marseille, France; ^2^Aix-Marseille Université, IRD, MEPHI, IHU Méditerranée Infection, Marseille, France; ^3^VBIC, INSERM U1047, Université de Montpellier, Service de Microbiologie et Hygiène Hospitalière, CHU Nîmes, Nîmes, France; ^4^Laboratoire de Microbiologie, Assistance Publique-Hôpitaux de Marseille, IHU Méditerranée Infection, Marseille, France

**Keywords:** metagenomic next generation sequencing, diagnosis, point-of-care laboratory, multiplex RT-PCR, culture, pathogen genome, antibiotic resistance, genotyping

## Abstract

Current routine diagnosis of community-acquired meningitis (CAM) by multiplex real-time polymerase chain reaction (RT-PCR) is limited in the number of tested pathogens and their full characterisation, requiring additional *in vitro* investigations to disclose genotype and antimicrobial susceptibility. We reviewed 51 studies published through December 2021 reporting metagenomic next generation sequencing (mNGS) directly applied to the cerebrospinal fluid (CSF). This approach, potentially circumventing the above-mentioned limitations, indicated 1,248 investigated patients, and 617 patients dually investigated by routine diagnosis and mNGS, in whom 116 microbes were detected, including 50 by mNGS only, nine by routine methods only, and 57 by both routine methods and mNGS. Of 217 discordant CSF findings, 103 CSF samples were documented by mNGS only, 87 CSF samples by routine methods only, and 27 CSF samples in which the pathogen identified by mNGS was different than that found using routine methods. Overall, mNGS allowed for diagnosis and genomic surveillance of CAM causative pathogens in real-time, with a cost which is competitive with current routine multiplex RT-PCR. mNGS could be implemented at point-of-care (POC) laboratories as a part of routine investigations to improve the diagnosis and molecular epidemiology of CAM, particularly in the event of failure of routine assays.

## Introduction

Community-acquired meningitis (CAM), a life-threatening condition with 40% mortality (Beaman, 2018), warrants a rapid diagnosis, possibly within 2 h at a point-of-care (POC) laboratory, providing identification of the causative pathogen among a repertoire of fewer than 20 pathogens (Leber et al., 2016; Launes et al., 2017; Soucek et al., 2019; Vincent et al., 2020), those most frequently encountered in the general population of patients, and supporting the initial medical decisions for hospitalisation and antibiotic treatment (Leber et al., 2016; Soucek et al., 2019; Tansarli and Chapin, 2020). Such a POC diagnosis, which is routinely made using nested or semi-nested polymerase chain reactions (PCRs), is prone to cross-contamination resulting in false-positive diagnoses (Boudet et al., 2019; Vincent et al., 2020). Current POC diagnosis also relies on the detection of a limited number of pathogens, and may thus miss microorganisms acting as life-threatening pathogens in selected populations, such as *Cryptococcus* in HIV-infected patients, in whom *Cryptococcus* most frequently causes fungal meningitis, with 223,000 new cases each year and 81% mortality (Ahn et al., 2017; Xing et al., 2019) and amoebas, such as *Acanthamoeba* (Greninger et al., 2015; Behera et al., 2016). The precise characterisation of such detected pathogens, including genotyping and anti-infectious susceptibility profiling, are pieces of medical information which are relevant for the immediate medical management of patients and contacts, and source tracing.

Metagenomic next generation sequencing (mNGS) directly applied to cerebrospinal fluid (CSF) emerged less than 10 years ago, in the specific context of healthcare-associated meningitis (Naccache et al., 2015), as an alternative to the laboratory diagnosis of CAM, potentially surpassing above-mentioned limitations of the current multiplex PCR approach, as discussed below (Table 1). However, no review papers have been published including recommendations and the routine diagnostic implications of this approach. To update existing knowledge about the direct diagnosis of CAM by metagenomics, we conducted a literature search for studies applying mNGS directly to CSF for the diagnosis of CAM.

**TABLE 1:** Metagenomics pipelines.

References	Country	Category	Sample type	Total patient	Sample preparation	DNA/RNA extraction	Microbial genome enrichment	Library preparation	Instrument	Software	Database
(Greninger et al., 2015)	California, United States	Case report	Fresh CSF	1	No	EZ1 Viral kit (Qiagen)	Turbo DNase (Ambion)	Nextera XT protocol (Illumina)	Illumina MiSeq	SURPI pipeline	NCBI GeneBank database
(Guan et al., 2015)	Tianjin, China	Prospective study	Frozen CSF	4	No	TIANamp Micro DNA Kit (TIANGEN BIOTECH)	Sigma-Aldrich WGA4 Kit (WGA)	Not available	BGISEQ-100 platform	Burrows-Wheeler Alignment, SoapCoverage software	Microbial Genome Databases
(Yao et al., 2016)	Beijing, China	retrospective study	Frozen CSF	3	No	TIANamp Micro DNA Kit (Tiangen Biotech)	No	Not available	BGISEQ-100	Burrows-Wheeler Alignment	Microbial Genome Database
(Joanna María et al., 2016)	Mexico	Case report	Frozen CSF	1	No	MagNA Pure LC 2.0 instrument, Total Nucleic Acid isolation kit (Roche)	TransPlex Whole Transcriptome Amplification kit WTA, (Sigma-Aldrich) and GenomiPhi V2 (Healthcare Life Sciences)	Roche 454 GS FLX single-end library	454 GS FLX Titanium system (Roche)	GS *de novo* Assembler version 2.6,	NCBI GenBank
(Kawada et al., 2016)	Nagoya, Japan	Prospective study	Fresh CSF	18	0.45-μm filter (Merck-Millipore, Temecula, CA)	QIAamp Viral RNA Mini kit (Qiagen)	Turbo DNase (Ambion, Darmstadt, Germany)	Nextera XT DNA Sample preparation kit and ScriptSeq v2 (Illumina)	Illumina MiSeq, Illumina HiSeq 2500	MEGAN5, CLC workbench	MEGABLAST
(Chiu et al., 2017)	California, United States	Case report	Fresh CSF	1	Not available	Not available	Not available	Not available	Illumina Miseq	SURPI+ computational pipeline	Microbial genome database
(Piantadosi et al., 2017)	Boston, United States	Case report	Fresh CSF	1	centrifugation 90 min/18,000 xg/4°C	QIAamp Viral Mini Kit (Qiagen)	TURBO DNase (Thermo Fisher)	Nextera XT	Illumina MiSeq	Kraken, *de novo* assembly	published computational pipeline viral-NGS
(Hu et al., 2018)	Nanjing, China	Case report	Fresh CSF	1	No	TIANamp Micro DNA Kit (TIANGEN BIO- TECH)	Sonication (Bioruptor Pico protocols)	end-repaired adaptation and PCR amplification (BGI, Tianjin, China)	BGISEQ-50 platform	Burrows-Wheeler Alignment	Microbial genome database
(Piantadosi et al., 2018)	Boston, United States	Case report	Fresh CSF	1	TURBO DNase (Thermo Fisher)	QIAamp Viral Mini Kit (Qiagen)	TURBO DNase (Thermo Fisher)	Nextera XT (Illumina)	Illumina MiSeq, HiSeq 2500	Kraken, Geneious version 8.1.7	NCBI GenBank Database
(Xing et al., 2019)	Beijing, China	Prospective study	Frozen CSF	12	Not available	Not available	Not available	Not available	BGISEQ-500/50 platform	Burrows-Wheeler Alignment	Microbial genome Database
(Tschumi et al., 2019)	Zurich, Switzerland	Case report	Fresh CSF	1	centrifugation and filtration	Not available	DNase treatment	Nextera XT (Illumina)	MiSeq	VirMet pipeline	NCBI GenBank database
(Saha et al., 2019)	Bangladesh	Retrospective study	Frozen CSF	91	Not available	Not available	Not available	Nextera XT (Illumina)	Illumina NovaSeq	IDseq bioinformatics pipeline	NCBI GenBank database
(Wang et al., 2019)	Beijing, China	Retrospective study	Frozen CSF	23	glass beads, vortexed 20 min, centrifuged at 8000 rcf	TIANamp Micro DNA Kit (TIANGEN BIOTECH)	No	end-repaired adaptation and PCR amplification (BGI, Tianjin, China)	BGISEQ-100	Mapping with WBA, BLAST	Microbial genome database
(Wilson et al., 2019)	California, United States	Retrospective study	Fresh CSF	204	Not available	Not available	Not available	Nextera XT (Illumina)	Illumina HiSeq	SURPI+ pipeline	NCBI GenBank database
(Zhang Y. et al., 2019)	Hebei, China	Case report	Fresh CSF	1	No	TIANamp Micro DNA Kit (Tiangen Biotech)	No	end-repaired adaptation and PCR amplification (BGI, Tianjin, China)	BGISEQ-100	Burrows-Wheeler Alignment	Microbial genome database
(Edridge et al., 2019)	Amsterdam, Netherlands	Retrospective study	Frozen CSF	45	Centrifugation TURBO DNase (Thermo Fisher)	Manually extracted Boom method	MseI (T?TAA; New England Biolabs)	VIDISCA library preparation	Ion PGM System	Taxonomer, CodonCode Aligner (version 6.0.2)	UBLAST
(Zhang X. X. et al., 2019)	Beijing, China	Retrospective study	Fresh CSF	135	No	TIANamp Micro DNA Kit (DP316, Tiangen Biotech, Beijing, China).	No	BGISEQ-500 standard protocol	BGISEQ-500 sequencing	Burrows-Wheeler Alignment	Microbial genome database
(Miller et al., 2019)	California, United States	Retrospective study	Fresh CSF	95	FastPrep-24 bead beater (MP Biomedicals)	EZ1 Virus Mini Kit v2.0 (Qiagen)	NEB Microbiome Enrichment Kit (New England Biolabs) Turbo DNAse (Thermo-fisher)	Nextera XT DNA Library Prep Kit (Illumina)	Illumina HiSeq	SURPI+ pipeline	GenBank reference database
(Eibach et al., 2019)	Agogo, Ghana	Retrospective study	Frozen CSF	70	No	MagMAX Viral RNA Isolation Kit (Life Technologies)	No	BGISEQ-500 standard protocol	Illumina MiSeq	CLC workbench, Trinity v2.6.6, Geneious v11, DIAMOND v0.9.6	NCBI GenBank database
(Chen et al., 2020)	Yangzhou, China	Case report	Fresh CSF	4	Centrifugation	TIANamp Micro DNA Kit (TianGen Biotech)	Ultrasonicator (Covaris)	VAHTS Universal DNA Library Prep Kit for Illumina V3 kit	Illumina NextSeq500	Burrows-Wheeler Alignment	Microbial genome database
(Lan et al., 2020)	Hunan, China	Case report	Fresh CSF	1	No	TIANGEN DNA Mini kit DP316 (Tiangen Biotech, Beijing, China)	No	Not available	Illumina NextSeq	Burrows-Wheeler Alignment	Microbial genome database
(Zhang et al., 2020)	Hunan, China	Case report	Fresh CSF	1	Not available	Not available	Not available	Not available	Not available	Not available	Not available
(Xing X. W. et al., 2020)	China	Prospective study	Frozen CSF	213	Glass beads, vortex	Not available	Not available	Not available	BGISEQ-500	Burrows-Wheeler Alignment	Microbial genome database
(Yan et al., 2020)	Shanghai, China	Prospective study	Frozen CSF	51	Glass beads, vortex 2800-3200RPM for 30 min	TIANamp Micro DNA Kit (TIANGEN BIOTECH)	No	end-repaired adaptation and PCR amplification (BGI)	BGISEQ-50 platform	Burrows–Wheeler Alignmen	Microbial Genome Databases
(Wang et al., 2020)	Wenzhou, China	Case report	Fresh CSF	1	Not available	Not available	Not available	Not available	BGISEQ platform	Burrows-Wheeler Alignment	Microbial Genome Databases
(Wu et al., 2020)	Shanghai, China	Case report	Fresh CSF	1	Not available	Not available	Not available	Not available	BGISEQ platform	Burrows-Wheeler Alignment	Microbial Genome Databases
(Manso et al., 2020)	London, United Kingdom	Prospective study	Fresh CSF	12	Filtration and Turbo DNAse treatment	PureLink Viral RNA/DNA Mini Kit (Invitrogen)	No	Nextera XT DNA library prep kit (Illumina)	Illumina MiSeq	Trimmomatic v0.39, PRINSEQ, mapping with PALADIN, BWA MEM	In-house database comprising the RefSeq viral protein sequences from NCBI
(Carbo et al., 2020)	Leiden, Netherlands	Prospective study	Frozen CSF	41	No	MagNApure 96 DNA and Viral NA Small volume extraction kit (ROCHE)	SpeedVac vacuum con- centrator (Eppendorf), SeqCap EZ Hypercap probes (Roche)	NEBNext Ultra II Directional RNA Library prep kit (New England Biolabs)	Illumina NovaSeq6000	Illumina data analysis pipeline RTA3.4.4 and bcl2fastq v2.20, Genome Detective version 1.111	An index database constructed from NCBI
(Leon et al., 2020)	Barcelona, Spain	Retrospective study	Frozen CSF	20	No	Direct-zol RNA MicroPrep with TRI reagent (Zymo Research)	Depletion of Abundant Sequences by Hybridization (DASH)	NEBNext Ultra II Directional RNA Library prep kit (New England Biolabs)	Illumina HiSeq 4000 instrument	Geneious version 10.2.3, SPAdes version 3.10.0, MUSCLE, MAFFT.	Local database
(Solomon et al., 2021)	Boston, United States	Case report	Frozen CSF	1	Not available	Not available	Not available	Not available	Illumina HiSeq 2500	SURPI+ pipeline	NCBI GenBank database
(Li et al., 2021)	Sydney, Australia	Prospective study	Frozen CSF	18	No	RNeasy plus universal kit (QIAGEN)	No	Trio RNA-Seq kit (NuGEN Technologies, United States) was	Illumina NovaSeq platform	Blastn, Blastx, Diamond, Megahit	NCBI GenBank databases,
(Zhan et al., 2021)	Hangzhou, China	Case report	Fresh CSF	1	Not available	Not available	Not available	Not available	BGISEQ-500 sequencing	Not available	Not available
(Zhou et al., 2021)	Hunan, China	Case report	Fresh CSF	1	No	Not available	No	Nexter XT	NextSeq 550	Kraken	In-house database
(Mao et al., 2021)	Guangzhou, China	Case report	Frozen CSF	1	Not available	TIANamp Magnetic DNA Kit (Tiangen)	Not available	PACEseq mNGS (Hugobiotech)	NextSeq 550	Burrows-Wheeler Alignment	Microbial genome database
(Zhang et al., 2021a)	Hunan, China	Case report	Fresh CSF	1	Not available	Not available	Not available	Not available	Not available	Not available	Not available
(Huang et al., 2021)	Guangzhou, China	Case report	Fresh CSF	1	centrifugation 5 min at 15,000 rpm	magnetic beads [Sagene, Guangzhou, CHINA]	No	Nexter XT	Illumina NextSeq 550 DX	Not available	Not available
(Xing N. et al., 2021)	Hebei, China	Retrospective study	Fresh CSF	7	Not available	Not available	Not available	Not available	BGISEQ-500 sequencing	Burrows-Wheeler Alignment	Microbial genome database
(Erdem et al., 2021)	Ohio, United States	Prospective study	Frozen CSF	37	No	QIAamp Viral RNA Mini Kit (Qiagen)	No	TruSeq Universal kit (Illumina)	Illumina HiSeq4000	FastQC, Cutadpat and PRINSEQ tools, Bowtie2 mapper 2.0.6, CDHIT tool. *de novo* assembled using MIRA (v 4.0), MegaBLAST	NCBI GeneBank database
(Xing X.-W. et al., 2021)	China	Case report	Frozen CSF	1	Not available	Not available	Not available	BGISEQ-500 standard protocol	BGISEQ 50 MGI DNBSEQ	Burrows-Wheeler Alignment	Microbial genome database
(Yin et al., 2021)	Jiangsu, China	Retrospective study	Frozen CSF	1	No	TIANamp Magnetic DNA Kit (Tiangen)	No	KAPA Hyper Prep Kit (KAPA Biosystems) according	Illumina NextSeq 550Dx	Trimmomatic v.0.36 software, Bowtie2 software. Kraken 2 software	Microbial genome database
(Zeng et al., 2021)	Changsha, China	Case report	Fresh CSF	1	Not available	Not available	Not available	PACEseq mNGS test (Hugobiotech)	Illumina NextSeq 550	Not available	Not available
(Guan et al., 2021)	Riyadh, Saudi Arabia	Case report	Frozen CSF	1	No	DNeasy Blood and Tissue Kits (QIAGEN)	No	NuGEN Ovation Ultralow Library System V2 (NuGEN)	Illumina HiSeq 4000, Illumina iSeq 100	MEGAHIT assembler v1⋅1 ⋅4, BBmap with 0 ⋅98, Metabat v2 ⋅12 ⋅1, Spades	NCBI GenBank database
(Zhang et al., 2021b)	Zhejiang, China	Case report	Fresh CSF	1	Not available	Not available	Not available	Nexter XT	Illumina NextSeq	Kraken	Kraken microbial database
(Piantadosi et al., 2021)	Boston, United States	Prospective study	Fresh CSF	68	No	QIAamp viral RNA minikit (Qiagen)	NEBNext microbiome DNA enrichment kit (New England BioLabs)	Nextera XT DNA library prep kit (Illumina)	Illumina MiSeq	KrakenUniq, BLAST	NCBI GenBank database
(Morsli et al., 2021d)	Marseille, France	Case report	Fresh CSF	1	Proteinase K 20 min	QIAamp Viral RNA Mini Kit solutions (Qiagen)	Turbo DNase (Thermo Fisher) and	Nextera XT V2	Illumina iSeq 100	Spade, BLAST, CLC workbench	NCBI GenBank database
(Fan et al., 2021)	Guangzhou, China	Retrospective study	Frozen CSF	11	Not available	Not available	Not available	Not available	BGISEQ platform	Burrows-Wheeler Alignment	Microbial Genome Databases
(Morsli et al., 2021c)	Marseille, France	Case report	Fresh CSF	1	Proteinase K 20 min	Virus Mini Kit v2.0 (Qiagen)	Spiked primer enrichment	Nextera XT V2	Illumina Miseq, iSeq 100	Spade, BLAST, CLC workbench	NCBI GenBank database
(Morsli et al., 2021b)	Marseille, France	Case report	Fresh CSF	1	Proteinase K 20 min	EZ1 DNA Kit (Qiagen)	No	Oxford Nanopore MinION library preparation	Oxford Nanopore MinION	EPI2ME, Kraken-2, Pavian	NCBI GenBank database
(Morsli et al., 2021a)	Marseille, France	Case report	Fresh CSF	1	Proteinase K 20 min	EZ1 DNA Kit (Qiagen)	No	Oxford Nanopore MinION library preparation	Oxford nanopore MinION, Illumina iSeq	EPI2M2, Spades, CLC genomic workbench	NCBI GenBank database
(Gao et al., 2021)	Beijing, China	Prospective study	Fresh CSF	38	centrifugation 10 min at 13,000 rpm	QIAamp DNA Microbiome Kit (Qiagen)	NEBNext microbiome DNA enrichment kit (New England BioLabs)	Nextera XT kit v2 Ion Torrent end-repair library	Illumina MiSeq, Ion Torrent Proton	Burrows-Wheeler alignment was	Microbial genome databases
(Morsli et al., 2022)	Marseille, France	Case report	Fresh CSF	1	Proteinase K 20 min	EZ1 DNA Kit (Qiagen)	No	Oxford Nanopore MinION library preparation	Oxford Nanopore MinION	EPI2ME and CLC Genomics Workbench, software	NCBI GenBank database

## Methods

### Literature Search

**FIGURE 1:**
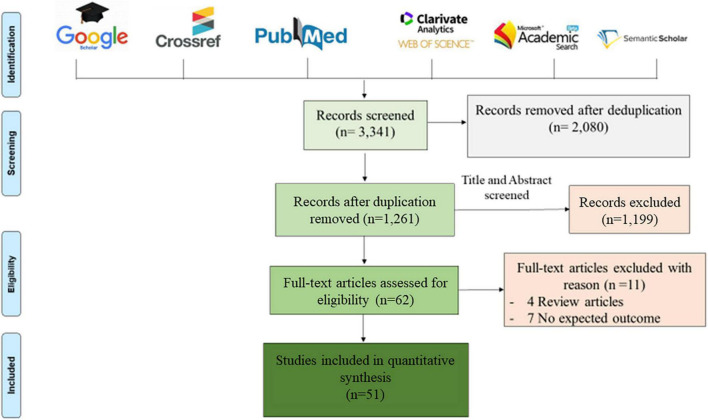
Prisma diagram of eligible articles recorded after removing duplications. Six bibliographic databases were reviewed using the following key words: “metagenomic,” “diagnosis,” “meningitis,” “encephalitis,” “next generation sequencing,” and “cerebrospinal fluid,” used alone and/or in combination. Only 51 articles were eligible for this review, after screening according to the inclusion criteria described above.

This study was conducted in accordance with the Preferred Reporting Items for Systematic Reviews and Meta-Analyses (PRISMA) guidelines (Moher et al., 2015). A systematic bibliography search was conducted on PubMed, Google Scholar, Web of Science, Microsoft Academic, Crossref and Semantic Scholar databases for studies published in English between January 1, 2015 and December 31, 2021 and related to the diagnosis of CAM by metagenomic NGS. Duplicate studies were removed in a first screening and the remaining papers were further screened based on title and abstract, according to the eligibility criteria. After full-text screening, only studies that met the inclusion criteria were included in this review, using the keywords: “metagenomic,” “diagnosis,” “meningitis,” “encephalitis,” “next generation sequencing,” and “cerebrospinal fluid.” These keywords were used in combination to perform an exhaustive search, as presented in Figure 1.

### Eligibility and Data

Studies with the following criteria were included in this review: Studies using mNGS-CSF for: (1) case reports; (2) prospective series; (3) retrospective series for non-routinely detected pathogens” (Table 1 and Supplementary Data). Review articles, studies performed on animals, non-clinical studies, and benchmarking studies were not included. Data extracted from selected studies included: first author name and year of publication, authors’ country, nucleic acid extraction method including commercially available kits, mNGS platform and sequencer instrument, pipeline data analysis and software, and reference database used for pathogen genome detection of identified microorganisms. Included were the total number and discordance/agreement of mNGS data with data yielded by routinely used methods, including molecular tests and culture. These data were reviewed and extracted after validation of the inclusion criteria by the authors.

## Results

### Included Studies

Bibliographic searches identified 3,341 articles and, following the Prisma diagram (Figure 1), 2,080 articles were removed after deduplication and 1,199 additional articles were removed by title and abstract screening. From the 62 remaining articles, 11 articles were removed after full-text reading, including four review articles and seven non-relevant articles, resulting in 51 articles analysed in this review.

### Study Characteristics

#### Participants

**FIGURE 2:**
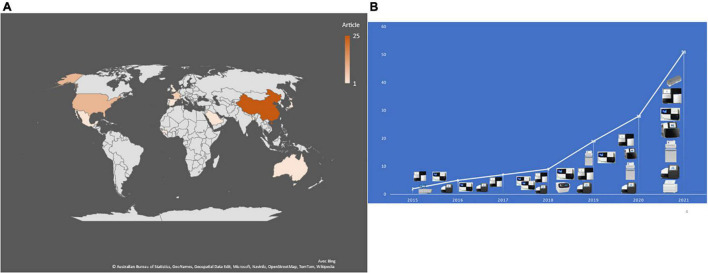
**(A)** Number and geographical distribution of publication records after duplication removed. **(B)** Publication chronology according to the used sequencing technologies.

In 2015, the first reported application of mNGS directly on CSF (and brain biopsy) enabled the post-mortem diagnosis of Astrovirus-related encephalitis in an immunocompromised patient (Naccache et al., 2015). A 7-year review of publications indicated only nine studies published between 2015 and 2018, ten in 2019 and 42/51 (82%) publications between 2019 and 2021, including 22 (43%) publications in 2021 (Figure 2). As for the geographic origin of published studies, 28 (56%) were published in Asia, including 25 (49%) from Chinese laboratories (Table 1), one each from Saudi Arabia (Guan et al., 2021), Bangladesh (Saha et al., 2019), and Japan (Kawada et al., 2016) (6.1%); nine in the United States (Table 1); one in Mexico (Joanna María et al., 2016); five in France (10%) (Tabel 1); two in Netherlands (Edridge et al., 2019; Carbo et al., 2020); and one each in Switzerland, the United Kingdom, and Spain (Tschumi et al., 2019; Leon et al., 2020; Manso et al., 2020). One study was also published in Guinea and another in Australia (Eibach et al., 2019; Li et al., 2021; Figure 2).

### Workflow

#### Nucleic Acid Procedures

Only CSF investigations [apart from one post-mortem brain biopsy in the founding study (Naccache et al., 2015)] were reviewed in this study. Depending on the prospective or retrospective nature of the investigations, fresh CSF was used in 63% of studies and frozen CFS in 37% of studies. DNA and RNA were manually extracted in 44% of studies, automatic extraction was performed in 20%, and 36% of studies did not report on the nucleic acid extraction method. The sample pre-treatment reported in 18/51 (35%) studies included centrifugation (∼15,000 *g*), filtration and vortexing in 12 studies, DNase treatment in four studies (Piantadosi et al., 2018; Edridge et al., 2019; Manso et al., 2020; Morsli et al., 2021d) and proteinase K treatment in five studies (Table 1; Guan et al., 2015; Miller et al., 2019; Carbo et al., 2020; Manso et al., 2020; Morsli et al., 2021a,c; Piantadosi et al., 2021), while no pre-treatment was performed in 15 studies and no such information was provided in 16 studies (Table 1). Extracted nucleic acids (retro-transcribed RNA and DNA) were always quantified using either a Qubit fluorometer and a Qubit DNA and RNA High Sensitivity Assay Kit (Life Technology, United States) (Table 1; Guan et al., 2015; Miller et al., 2019; Carbo et al., 2020; Manso et al., 2020; Morsli et al., 2021a,c; Piantadosi et al., 2021), or NanoDrop spectrophotometer (Thermo Fisher Scientific, United States) (Li et al., 2021). Optimisation in the human-to-microbial ratio of DNA was achieved by intermediate microbial genome enrichment or human genome depletion (Table 1). DNase treatment was used to reduce human DNA in extracted RNA for mNGS investigations of RNA-viruses (Table 1; Guan et al., 2015; Miller et al., 2019; Carbo et al., 2020; Manso et al., 2020; Morsli et al., 2021a,c; Piantadosi et al., 2021), and bead-based capture kits were also used to remove human DNA (Miller et al., 2019; Leon et al., 2020; Gao et al., 2021; Piantadosi et al., 2021). Further, microbial genomes could be enriched by specific primer amplification for whole genome investigations (Table 1). cDNA and double-stranded synthesis were needed prior to library preparation when RNA was investigated (Table 1), and in the case of RNA genome enrichment, an RT-one-step protocol was followed by reverse-transcription and pathogen genome amplification (Morsli et al., 2021c). In two studies, DNA was mechanically broken prior to library construction (Hu et al., 2018; Zhang X. X. et al., 2019).

#### Next Generation Sequencing Library Preparation and Sequencing

**FIGURE 3:**
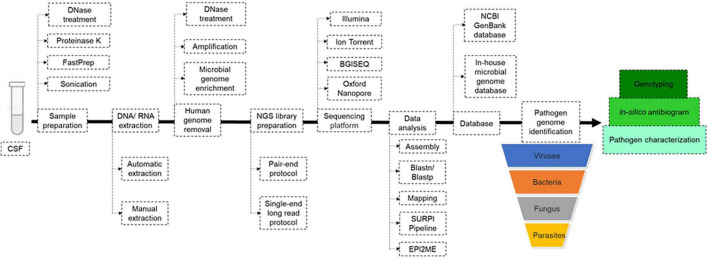
Metagenomic next generation sequencing (mNGS) workflow. Upon reception of the cerebrospinal fluid (CSF), mechanical and enzymatic sample pre-treatment was performed before manual or automatic DNA/RNA extraction. Before mNGS library preparation, the human genome was removed with or without microbial genome enrichment, then mNGS libraries were prepared following pair-end or single-end long read protocol, depending on the sequencing platform. Pathogen genome identified directly by blast against NCBI GenBank and/or local microbial genome database downloaded from GenBank, using in-house pipelines. *In-silico* antibiogram and genotyping were performed depending on the nature of the identified pathogenic agent.

Three main protocols were used for mNGS library preparation (Table 1 and Figure 3). Illumina pair-end protocols and reagents were used for mNGS library preparation in 19/51 (37%) studies, including Nextera XT DNA Library Prep Kit in 16/51 (31%) and one each with the TruSeq Universal kit (Erdem et al., 2021) and VAHTS Universal DNA Library Prep Kit (Chen et al., 2020). Standard BGISEQ pair-end protocols were used in 6/51 studies. Two studies used the PACEseq mNGS test (Hugobiotech, Beijing, China) (Mao et al., 2021; Zeng et al., 2021), two studies used VIDISCA and Ion Torrent end-repair library (Edridge et al., 2019; Gao et al., 2021), and one study each used the NEBNext Ultra II Directional RNA Library prep kit (New England Biolabs, Ipswich, MA, United States) (Carbo et al., 2020; Leon et al., 2020), the KAPA Hyper Prep Kit (Kapa Biosystem, Potters Bar, United Kingdom) (Yin et al., 2021), the NuGEN Ovation Ultralow Library System V2 kit (NuGEN Technologies, United States) (Guan et al., 2015) the Trio RNA-Seq kit (NuGEN Technologies) (Li et al., 2021), and the Roche 454 GS FLX single-end library (Roche Diagnostics, Branford, CT, United States) (Joanna María et al., 2016). In three studies, the mNGS library was constructed following the single-end Oxford Nanopore library preparation protocol (Morsli et al., 2021a,b, 2022). Finally, the library preparation protocol and kits were not reported in 14 studies (Table 1 and Figure 3). mNGS libraries were sequenced using Illumina sequencers in 30/51 (59%) studies, MiSeq platform in 11/30, HiSeq platform in 8/30, Nextseq platform in 6/30, iSeq 100 platform in 4/30, and 3/30 studies used the NovaSeq platform. BGISEQ platforms were used in 12/51 (24%) of studies (Table 1), including BGISEQ-50, 100, and 500 platforms, while three studies used the Roche 454 GS FLX Titanium system platform, Ion PGM System platforms and Ion Torrent Proton Sequencer (Life Technologies, United States), respectively (Joanna María et al., 2016; Edridge et al., 2019; Gao et al., 2021). Real-time sequencing with the Oxford Nanopore MinION sequencer was used in three studies (Morsli et al., 2021a,b, 2022) and no information about the sequencer was reported in five studies (Table 1). The use of Nanopore technologies, i.e., single-long-read sequencing platforms combined with direct read blasting against the NCBI GenBank database using EPI2ME online software^1^ or against an internal database using in-house pipelines, allowed for pathogen identification within the first minutes of sequencing (Morsli et al., 2021a). Furthermore, all additional pieces of information such as bacteria profiling and genotyping, was obtained in less than 6 h (Morsli et al., 2021a,b, 2022).

#### Sequence Data Analysis

Next generation sequencing data quality was controlled using FastQC (Erdem et al., 2021; Yin et al., 2021), and the human genome could be removed by mNGS reads mapping to the *Homo sapiens* reference genome (*hg*19) using Burrows-Wheeler Alignment (BWA) in 40% of studies. Non-mapped reads could be assembled using Spades and *de novo* assembly or directly against the reference genome using the BWA and CLC Genomic workbench (Table 1). For an exhaustive pathogen genome investigation, mNGS data were blasted [BLAST: Basic Local Alignment Search Tool (nih.gov)] against the NCBI GenBank database or against an in-house microbial genome database, regardless of the sequencing technology, yet with different timing as for Illumina and Nanopore technologies, as mentioned above (Table 1). The sequence-based ultra-rapid pathogen identification computational pipeline (SURPI) was a powerful method for pathogen identification, combining blast and mapping of both pathogen genome and protein encoding sequences directly from mNGS outputs (Greninger et al., 2015; Miller et al., 2019). When only one specific aetiology was investigated, a reference database constructed with such specific pathogen genomes and proteins could be used for its accurate identification (Piantadosi et al., 2017; Manso et al., 2020). Depending on the previous pathogen identification, a specific pathogen genome was extracted directly as consensus sequences in FASTA files, after mapping to the reference genome directly from CLC and BWA software for further analysis (Table 1 and Figure 3). Virus genotyping based on hit-blast identified strains could be confirmed by phylogenetic analysis based on sequence similarity level, while bacteria genotyping could be predicted online by Multi-Locus Sequence Typing online platform (Figure 3). Also, the *in silico* antibiotic resistance pattern could be predicted by aligning bacterial genome sequences against online databases of antibiotic-resistance encoding genes (Table 1; Guan et al., 2015; Miller et al., 2019; Carbo et al., 2020; Manso et al., 2020; Morsli et al., 2021a,c; Piantadosi et al., 2021), mostly represented by ResFinder on the Centre for Genomic Epidemiology server (Bortolaia et al., 2020; Morsli et al., 2021a,b, 2022).

#### Pathogen Detection and Characterisation

**FIGURE 4:**
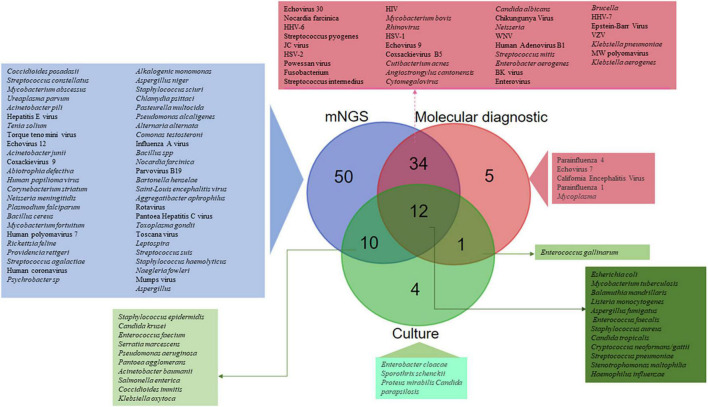
SAM pathogens identified by routine molecular diagnosis, culture, and metagenomic next generation sequencing (mNGS). A total of 116 different microorganisms were identified, including 50 (43.1%) only by mNGS, 43 by both routine molecular diagnosis and mNGS, 12 by culture, mNGS and routine molecular diagnosis, 10 by culture and mNGS, five only by routine molecular diagnosis, four only by culture, and one only by culture and routine molecular diagnosis.

Of the 51 studies analysed, a total of 1,248 CSF samples collected from CAM patients were investigated in parallel by mNGS reference and routine molecular diagnosis and culture. Routine methods and mNGS yielded concordant results in 1,031 (82.6%) CSF samples, including no pathogen documentation in 566 CSF samples and concordant identification in 465 CSF samples. Discordant results were observed in 217 (17.4%) CSF samples, including 87 CSF samples documented by routine methods only, 103 CSF samples documented by mNGS only, and 27 CSF samples in which documented microorganisms differed from routine methods and mNGS. Altogether, the 116 detected microorganisms included 63 bacteria, 38 viruses, 10 fungi, and five parasites. mNGS detected 106/116 (91.4%) different microorganisms, including 50 (43.1%) detected by mNGS only, whereas 56 microorganisms were detected by both routine methods and mNGS. In addition, five microorganisms were detected by routine molecular diagnosis only, four microorganisms by culture only and one *Enterococcus gallinarum* strain was diagnosed by routine molecular diagnosis and culture (Figure 4). Further, there was concordant documentation of 56 different pathogens, including 30 bacterial pathogens, 19 viral pathogens, six fungi and one *Balamuthia mandrillaris* (*B. mandrillaris*) as the only amoeba detected in a CSF sample (Table 1 and Figure 4). Of the 217 CSF samples in which mNGS investigation yielded results discordant with the routine method, mNGS conclusively found a pathogen in 38/103 CSF samples in which routine investigations found no pathogen, as reported in 25/51 studies: mNGS over-detected RNA viruses, including *Human rhinovirus* and *Human coronavirus* (Li et al., 2021), *Coxsackievirus* 9 and Mumps virus (Kawada et al., 2016), *Saint-Louis Encephalitis virus* (Chiu et al., 2017), *Powassan Virus* (Piantadosi et al., 2018), *Toscana virus* (Tschumi et al., 2019), *Jamestown Canyon virus* (Solomon et al., 2021), Enterovirus A71 (Leon et al., 2020), and *Hepatitis E virus* (Carbo et al., 2020), while only HSV-1, HHV-6, and EBV were identified as DNA viruses (Zhang Y. et al., 2019; Carbo et al., 2020). Bacterial pathogens documented in 11% of these cases included *Listeria monocytogenes* in four cases (Yao et al., 2016; Lan et al., 2020), *Ureaplasma parvum* in three cases (Wang et al., 2020; Xing X.-W. et al., 2021; Zhan et al., 2021) and one case each was detected of *Klebsiella pneumoniae* (Zeng et al., 2021), *Pasteurella multocida* (Morsli et al., 2022), *Enterococcus faecalis* (Zhang et al., 2021a), *Nocardia farcinica* (Zhang et al., 2021b), *Streptococcus suis* (Zhang et al., 2020), and *Psychrobacter* sp. (Joanna María et al., 2016). *Toxoplasma gondii, B. mandrillaris*, and *Naegleria fowleri* parasite species were identified in four patients (*N*. *fowleri* in two patients) in whom routine diagnostic methods completely missed *N. fowleri* (Hu et al., 2018; Wu et al., 2020; Guan et al., 2021; Huang et al., 2021), while *Coccidioides posadasii* was the single fungal infection exclusively detected by mNGS in one patient with no previously documented infectious meningitis (Mao et al., 2021; Supplementary Data; Figure 4).

#### Limitations

A review of published data indicated that in 4/217 cases, mNGS failed to detect a pathogen when the pathogen had been detected by real-time polymerase chain reaction (RT-PCR) with a Ct > 37, mainly due to a low pathogen inoculum (Miller et al., 2019), as well as for a pathogen detected by immunodetection assay (Xing X.-W. et al., 2021). These limitations could be due to:

1) Storage conditions: storage of the CSF at temperature above -80°C may cause nucleic acid molecule damage. Moreover, lack of proper storage in the presence of RNase inhibitors, will likely result in RNA viruses undergoing some degradation.
2) Limited CSF volume affecting the DNA quantity used for NGS library preparation (Miller et al., 2019), especially for the Oxford Nanopore libraries, which require 1 μg of DNA (Morsli et al., 2021a,b).
3) A CSF leukocyte count (>10^3^/mL) increasing the ratio of human genome, which required microbial genome enrichment (Guan et al., 2015; Miller et al., 2019; Carbo et al., 2020; Manso et al., 2020; Morsli et al., 2021a,c; Piantadosi et al., 2021), and human genome depletion (Table 1). DNase treatment usually used to reduce human DNA in the extracted CSF can, therefore, generate false negative results, due to the degradation of the DNA pathogens (Edridge et al., 2019). Alternatively, one limitation is related to the elimination of human DNA prior tosequencing, such as CSF pathogens whose genome is partially or totally integrated into the human genome, such as the case of HHV-6 (Edridge et al., 2019). Library preparation and cross-contamination removal during sample preparation is essential to avoid any confusion of detection and interpretation, especially when working on several samples in the same run (Table 1; Guan et al., 2015; Miller et al., 2019; Carbo et al., 2020; Manso et al., 2020; Morsli et al., 2021a,c; Piantadosi et al., 2021). Depth, coverage, and sequence quality may be influenced by the sequencing platforms, as well as the kit used for library preparation, which is relative to the initial pathogen load in the CSF (Table 1; Guan et al., 2015; Miller et al., 2019; Carbo et al., 2020; Manso et al., 2020; Morsli et al., 2021a,c; Piantadosi et al., 2021), depending on the number of sequencing cycles for paired-end sequencing and the read size for single-end long read sequencing. Assembly and data analysis mainly depend on the quantity and quality of reads generated by sequencers (Morsli et al., 2021a,c,d). Pathogen identification is mainly based on in-house databases constructed with a limited number of pathogen genomes and species downloaded from the NCBI GenBank (Table 1) used for blast and mapping, which needs regular updating for the exhaustive identification of all possible microbial genomes detected in the CSF sample (Table 1). Performance analysis requires powerful software and pipelines to blast all mNGS data against the GenBank database, which is limited by cost and accessibility, such as the EPI2ME software used for real-time analysis of sequencing data generated by Nanopore Platforms (Morsli et al., 2021a,b, 2022).

## Discussion

Direct mNGS appears to be a relevant alternative diagnostic approach to simplex and multiplex RT-PCRs for the POC diagnosis of CAM. mNGS adds one-shot pieces of medically relevant information, including genotyping and antimicrobial susceptibility profile, in comparison to current PCR-based methods (Morsli et al., 2021a,b; Zhou et al., 2021). The suitability of this new approach should be evaluated in all laboratories already providing molecular detection of pathogen-genome sequences in the CSF of patients with meningitis, and as a new technique in new POC laboratories bypassing previous multiplex RT-PCR (Boudet et al., 2019; Vincent et al., 2020; Morsli et al., 2022). The basis for such an evaluation includes the flow of samples and cost of mNGS relative to multiplex PCR. Indeed, mNGS also provides an antimicrobial susceptibility profile to guide medical management of the patient (Morsli et al., 2021a,b, 2022) and genotyping to detect outbreaks and to guide source tracing compared to multiplex PCRs (Broberg et al., 2018). Such genotyping information may be medically relevant and associated with a particular prognosis of CAM (case of a few Enteroviruses) and certainly relevant to source tracing in the context of clustered cases and outbreaks. Compared to the limited number of the pathogens investigated by routine multiplex RT-PCR, mNGS identified 106 different microorganisms in CSFs from patients with meningitis, i.e., covering 91.4% of total microorganisms identified here. This allowed the detection of pathogen genomes in 38 non-routinely documented CSFs. In addition, mNGS can operate within infinitely greater open databases than those currently supporting available multiplex RT-PCR assays, which rely on fewer than 30 such entries, whereas genome sequence databases such as NCBI consist of more than 83,124 complete microbial genomes (including bacteria, viruses, fungi, and parasites), offering opportunities for the diagnosis of rare pathogens, as reviewed in this study.

Finally, a few current limitations of the mNGS approach are opportunities for improvements from the perspective of routine use. Most enzymes used in the above-described sequencing protocols were issued from cloning in competent bacteria such as *Escherichia coli* (*E. coli*) (Morsli et al., 2021a), meaning potential contamination by *E. coli* DNA and a risk of a false-positive diagnosis of *E. coli* meningitis. As an illustration, Nanopore sequencing results regularly consisted of *E. coli* DNA resulting from the recommended internal library control, and *Shigella* and T4 phage reads issued from repair and ligation enzymes (Morsli et al., 2021a,b, 2022). This limit could be overcome by replacing the *E. coli* control and using the human genome as an internal control, in line with our own practice. The quantity of data generated by sequencing was sometimes insufficient to support an accurate interpretation, requiring additional enrichment and human genome depletion steps to improve the sensitivity and specificity of detection of CAM causative agents (Miller et al., 2019; Carbo et al., 2020; Gao et al., 2021; Morsli et al., 2021c; Piantadosi et al., 2021). Moreover, sequence variation linked to high mutational levels, as observed in RNA viruses including Enterovirus (Piantadosi et al., 2017; Morsli et al., 2021d,c), with more than 300 genotypes (Isaacs et al., 2018), and obscuring the identification capability of the mNGS approach, further indicates the necessity for pathogen genome enrichment to achieve appropriate sequencing depth. This is usually limited by the CSF volume which makes it not possible to extract the DNA and RNA separately (Li et al., 2021). The sensitivity of mNGS depended on the nature of the causative pathogen, being lower than routine techniques for bacterial and fungal meningitis, at 73.3% and <85%, respectively (Xing X. W. et al., 2020; 2021). In contrast, mNGS appeared to be highly sensitive (>90%) in cases of mycobacterial and viral meningitis (Kawada et al., 2016; Miller et al., 2019; Xing X. W. et al., 2020; Zhang et al., 2021b). However, human genome depletion by DNase treatment can produce false-negative results in the case of RNA investigation, by degrading the pathogen DNA (Edridge et al., 2019; Wilson et al., 2019).

The mNGS approach is not currently standardised and remains in its infancy. Microbiologists may, therefore, wish to develop different strategies to investigate RNA and/or DNA pathogens, relying on available mNGS materials and reagents as well as mNGS platform, and in-house pipelines using local epidemiology-driven, specific pathogen genome databases (Figure 3). Finally, because this identification is database-dependent, attention should be given to curating and updating local databases (Ji et al., 2020; Carbo et al., 2021; Xing X.-W. et al., 2021). In contrast to routine tests based on nested and semi-nested PCRs generating up to 25% false-positive results (Boudet et al., 2019), the mNGS approach does not involve any prior amplification and no specific pathogen target, thus limiting the opportunity for such false-positives. Additional information about genotyping and *in silico* antibiotic susceptibility testing was added, even in cases of uncultured bacteria.

As a time and cost-effective approach, real-time mNGS could be implemented in POC laboratories for the diagnosis of CAM, especially with regards to undocumented meningeal disease (Morsli et al., 2022).

## Author Contributions

MM: literature search, data collection, data cleaning, data interpretation, validation, and writing of the manuscript. JL and MD: design of the study, data interpretation, validation, funding, critical review of the manuscript, coordination, and direction of the work. The data presented here were extracted by MM and validated by JL and MD after review. All authors have read and approved the manuscript.

## Conflict of Interest

The authors declare that the research was conducted in the absence of any commercial or financial relationships that could be construed as a potential conflict of interest.

## Publisher’s Note

All claims expressed in this article are solely those of the authors and do not necessarily represent those of their affiliated organizations, or those of the publisher, the editors and the reviewers. Any product that may be evaluated in this article, or claim that may be made by its manufacturer, is not guaranteed or endorsed by the publisher.

## Footnotes

1
https://epi2me.nanoporetech.com/
