# Supplementary figures and images for: Direct Metagenomic Diagnosis of Community-Acquired Meningitis: State of the Art
Source: Front Microbiol. 2022 Jul 5;13:926240. doi: 10.3389/fmicb.2022.926240 (PMC9294516; doi:10.3389/fmicb.2022.926240)

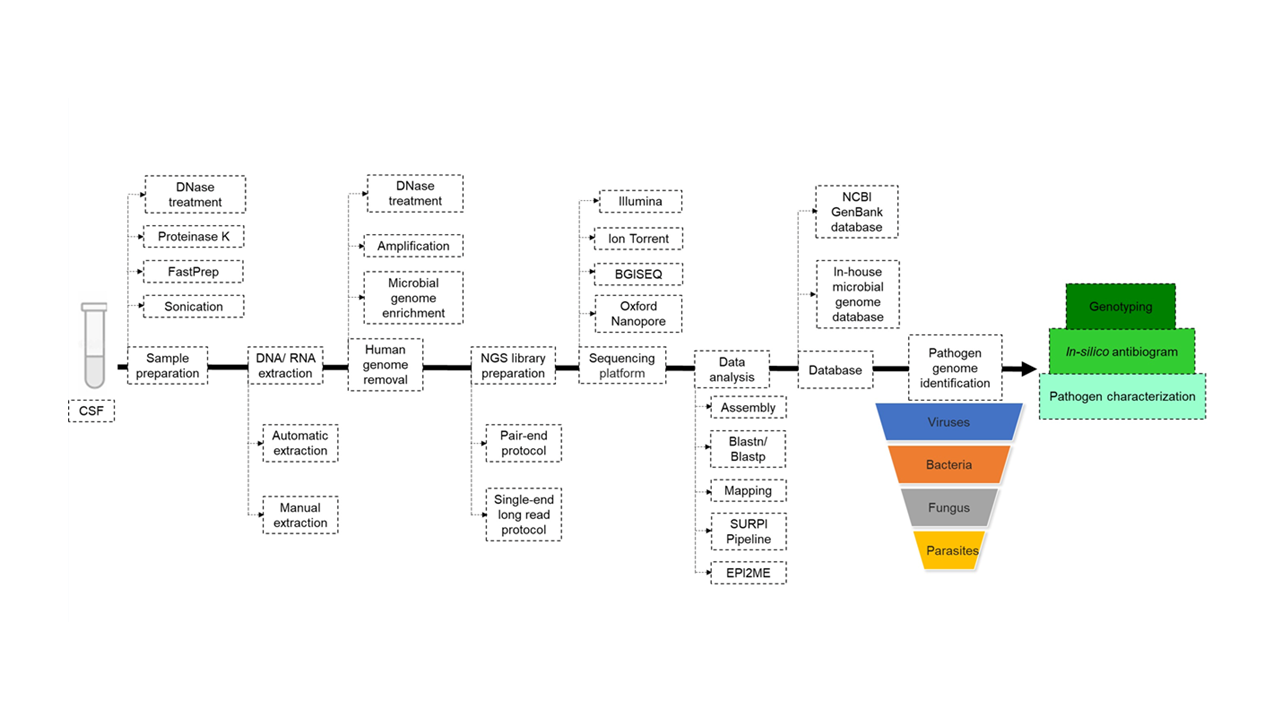

Supplement: Supplementary file 1 [file Image_1.TIF]

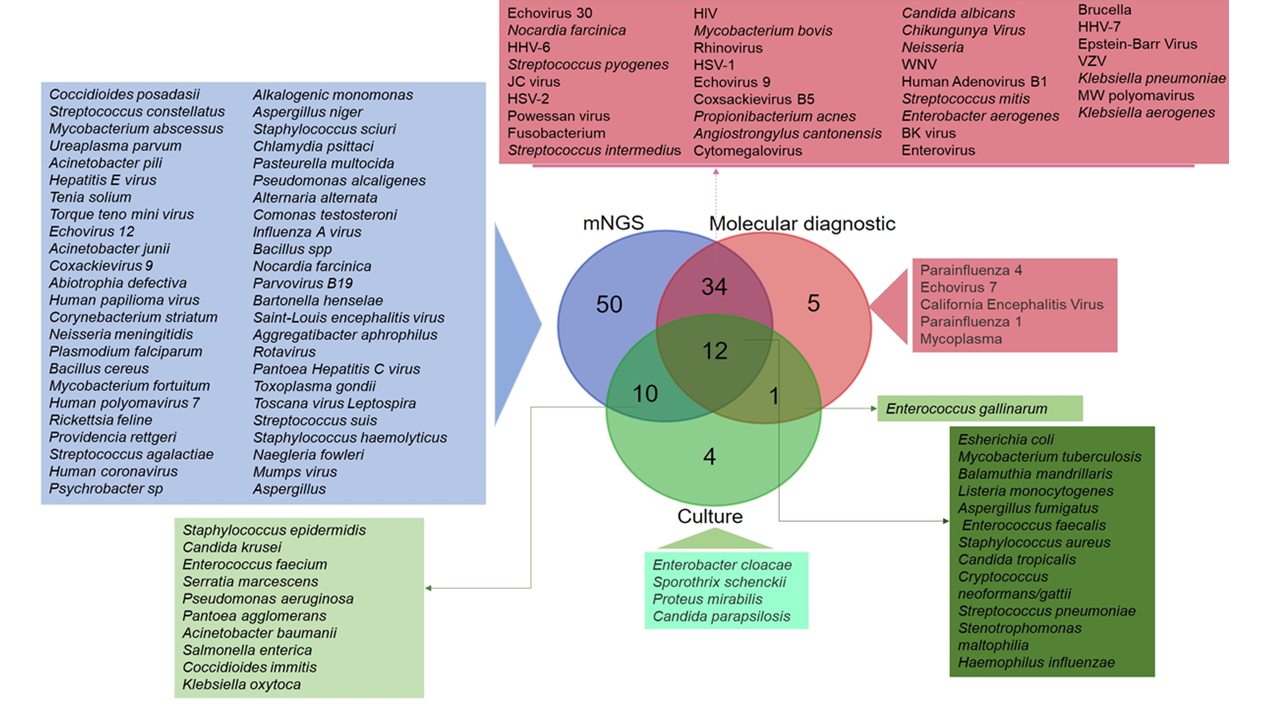

Supplement: Supplementary file 2 [file Image_2.TIF]
